# Modulation of Diacylglycerol-Induced Melanogenesis in Human Melanoma and Primary Melanocytes: Role of Stress Chaperone Mortalin

**DOI:** 10.1155/2019/9848969

**Published:** 2019-04-14

**Authors:** Renu Wadhwa, Ling Li, Rumani Singh, Jia Wang, Ran Gao, Nupur Nigam, Sandra Forestier, Nobuhiro Ando, Sunil C. Kaul

**Affiliations:** ^1^DAILAB, DBT-AIST International Center for Translational and Environmental Research (DAICENTER), National Institute of Advanced Industrial Science & Technology (AIST), Tsukuba 305-8565, Japan; ^2^KK Chanel Research and Technology Development Laboratory, 1-1-5, Yamate, Funabashi-Chiba 273-0045, Japan

## Abstract

Skin color/pigmentation is regulated through melanogenesis process in specialized melanin-producing cells, melanocytes, involving multiple signaling pathways. It is highly influenced by intrinsic and extrinsic factors such as oxidative, ultraviolet radiations and other environmental stress conditions. Besides determining the color, it governs response and tolerance of skin to a variety of environmental stresses and pathological conditions including photodamage, hyperpigmentation, and skin cancer. Depigmenting reagents have been deemed useful not only for cosmetics but also for pigmentation-related pathologies. In the present study, we attempted modulation of 1-oleoyl-2-acetyl-glycerol- (OAG-) induced melanogenesis in human melanoma and primary melanocytes. In both cell types, OAG-induced melanogenesis was associated with increase in enhanced expression of melanin, tyrosinase, as well as stress chaperones (mortalin and HSP60) and Reactive Oxygen Species (ROS). Treatment with TXC (trans-4-(Aminomethyl) cyclohexanecarboxylic acid hexadecyl ester hydrochloride) and 5/40 natural compounds resulted in their reduction. The data proposed an important role of mortalin and oxidative stress in skin pigmentation and the use of TXC and natural extracts for modulation of pigmentation pathways in normal and pathological conditions.

## 1. Background

Skin color, a well-established heritable trait, is highly influenced by environmental and endocrine factors [1, 2]. Upregulation of melanin in specialized melanin-producing cells is most often observed in response to environmental stresses and has been established to provide protection against UV-induced DNA damage [3]. On the other hand, oxidative stress and photodamage have been established as causes of skin cancer [3–5]. Sunscreens have emerged as an important daily consumer product to protect against UV-induced damage and pigmentation especially for white skin. The latter has been linked to high incidence of Dysplastic Naevi [6], high oxidative, and DNA damage stress [7]. At the same time, Vitiligo, a patchy loss of inherited skin color, is also characterized by high level of oxidative stress [8] suggesting that regulation of melanogenesis is an extremely important aspect of skin characteristics and response to the environmental stresses. The natural and synthetic reagents that modify these responses may hence possess high preventive and therapeutic potentials.

Mammalian melanogenesis is a highly complex process where oxidative stress plays an important role [9]. MITF transcription factor and KIT tyrosine kinase, major regulator of melanogenesis, have been shown to play critical role in regulation of mitochondrial membrane potential, cell proliferation, and survival [10–13]. Manipulation of pigmentation provides a model system for resolving the intricacies of melanogenesis, develops preventive, and therapeutic reagents for pigmentation associated diseases [14]. Achieving such goals are of high significance in cosmetic (skin whitening from beauty perspectives and skin tanning products for reducing the potential risk from skin cancer) and pharmaceutical (therapy for pigmentary diseases) industries. In view of this, we used OAG-induced melanogenesis [15, 16] in human skin-derived (melanoma and primary melanocytes) cells and investigated (i) the association of stress chaperones, mortalin, and HSP60 in melanogenesis and (ii) its modulation by TXC (trans-4-(Aminomethyl)cyclohexanecarboxylic acid hexadecyl ester hydrochloride), a derivative of an anti-inflammatory compound tranexamic acid (TXA). In contrast to TXA that is water-soluble and poorly penetrates the skin (hydrophobic characteristics), TXC (a cetyl ester compound) is hydrophobic and exhibits higher skin penetrating efficacy. TXC was shown to possess cyclohexane ring structure and a new type of amphiphile with the strong intermolecular interaction that results in formulation of unique self-assembly and bicontinuous alpha-gel contributing to excellent skin permeation [17]. TXC has been proposed as a multifunctional molecule that restricts production of hydroxyl radicals and inhibits multiple skin targets in pigmentation process [18]. It has been approved as a quasi-extract active ingredient by the Ministry of Health, Labor and Welfare of Japan, in 2009. However, its effect on human cell culture system has not been investigated. We used* in vitro* cell culture of human melanoma and primary melanocytes. Activation of melanogenesis by OAG in both cell types resulted in an upregulation of stress chaperones and ROS and downregulation of mitochondrial membrane potential. TXC inhibited OAG-induced pigmentation and reverted the molecular changes to a large extent. Similar attenuation of OAG-induced melanogenesis was observed in 5 out of 40 natural extracts. The data endorsed the role of stress chaperones in pigmentation and offered new extracts that warrant further studies on their functional characterization, molecular mechanisms of action, and efficacy for modulation of pigmentation pathways in normal and pathological conditions.

## 2. Material and Methods

### 2.1. Cell Culture and Viability Assay

Human skin melanoma (G361) cells and primary human melanocyte from Caucasian skin (PMC) were obtained from Japanese Collection of Research Bioresources (JCRB, Japan) and Kurabo Industries Ltd. (Osaka, Japan), respectively. G361 cells were cultured in McCoy's 5A medium (Life Technologies, Carlsbad, CA, USA) supplemented with 10% fetal bovine serum. PMC were cultured in Derma Life Basal Medium (Life Line Cell Technology, Carlsbad, CA). OAG (1-oleoyl-2-acetyl-glycerol) (Sigma, Japan) was dissolved in dimethylsulfoxide (DMSO) to obtain 10 mg/ml stock solution and added to the subconfluent (60-70% confluence) cells. After the initial dose response and toxicity assays for OAG, 30 *μ*g/ml for G361 melanoma and 15 *μ*g/ml for PMC were used. Cells were treated with TXC or other natural extracts (as indicated) at 60-70% confluency. Cell viability assay was performed using MTT (Life Technologies) and quantitative measurement of conversion of yellow MTT to purple formazan by mitochondrial dehydrogenases of viable cells. Statistical significance of the results was determined from 3-4 independent experiments including triplet or quadruplet sets in each experiment. Control and treated cells were observed under the microscope and photographed to record their morphology.

### 2.2. Melanin Content

G361 melanoma (2 X 10^3^/well) and primary human melanocytes (5 X 10^3^/well) were plated in 96-well dish. Cells were treated with OAG typically for 24 h. For melanin assay, the cells were incubated with 0.85 N KOH (100 *μ*l) with slow shaking at room temperature (RT) overnight. Melanin content was estimated by reading absorbance at 405 nm using a spectrophotometer (Tecan, Switzerland). Relative amount of melanin was calculated by using synthetic melanin (Sigma) as a standard in similar assays and normalized against protein content.

### 2.3. Tyrosinase ELISA

Cells were plated in 96-well plates (NUNC-IMMUNO, Maxisorp) and cultured until (24 or 48 h) they attached well to the surface. Control and OAG-treated cells were lysed with RIPA buffer (Thermo Fisher Scientific Inc., IL) and stored in −80°C until further assay. Protein concentration was estimated using Pierce BCA Protein Assay Kit (Thermo Scientific, IL, USA). Equal amounts of the protein from control and treated cells were diluted in coating buffer (0.1 M sodium bicarbonate pH 9.6 with 0.02% sodium azide) and incubated in plates at 4°C for overnight. Plates were washed with washing buffer (PBS, 0.5% Tween, pH 7.4) by shaking for 10 min (twice). Cells were incubated in blocking buffer (1% bovine serum albumin and 0.02% NaN3, pH 7.4) at 4°C (overnight) followed by two washings in washing buffer (10 min each). Cells were then incubated with antityrosinase polyclonal antibody (M-19)-R (1:5000 dilution in blocking buffer) at RT for 1 h followed by three washings in the washing buffer. Cells were incubated with secondary antibody (Alkaline phosphatase-goat anti-rabbit IgG) (1:1000 dilution in blocking buffer) for 45 min followed by five washings in washing buffer. Cells were then incubated with AP substrate pNPP (p-Nitrophenyl phosphate) (pNPP, 1 mg/ml) (Pierce) in substrate buffer (50 mM NaHCO_3_ and 10 mM MgCl_2_ 6H_2_O, pH 9.8) at RT for 30 min, followed by measurement of absorbance at 405 nm.

### 2.4. Immunofluorescence, Mitochondrial Membrane Potential and Reactive Oxygen Species (ROS) Assays

Cells were plated on coverslips placed in a 12-well culture plate. After the indicated treatments, cells were fixed in prechilled methanol: acetone (1:1) for 5-10 min followed by washing with PBS-T (PBS with 0.2% Triton X-100) and incubation with primary antibodies at 4°C overnight. Antibodies used are anti-melanosome (HMB45 recognizes 10-kD segment of a sialylated glycoconjugate) (Dako), anti-mortalin [14], anti-CARF [19], anti-HSP70 (SMC-100, StressMarq), anti-HSP60 (SPC-105C/D, StressMarq), anti-tyrosinase (H-109, Santa Cruz), anti-NFkB (sc-109, Santa Cruz), and anti-*γ*H2AX (07-627, Millipore). Cells were washed extensively with PBS-T followed by incubation with fluorochrome-conjugated secondary antibodies (Alexa-488-conjugated goat anti-rabbit, anti-mouse or Alexa-594 conjugated goat anti-rabbit or anti-mouse (Molecular Probes)). Cells were washed extensively with PBS-T and processed for imaging.

Mitochondrial membrane potential was determined in control and treated cells by using JC-1 Assay Kit (Cell Technology Inc., USA) that utilizes a unique cationic dye (5,5,6,6′-tetrachloro-1,1′,3,3′-tetraethylbenzimidazolylcarbocyanine iodide) to signal the loss of mitochondrial membrane potential. While healthy mitochondria are stained bright red, the collapse of mitochondrial membrane potential is seen as green fluorescence, as described earlier [20].

Reactive Oxygen Species (ROS) were detected by fluorescent staining using the Image-iT™ LIVE Green Reactive Oxygen Species (ROS) Detection Kit (Molecular Probes, Eugene, OR). Images, in all cases, were captured on a Zeiss Axiovert 200M microscope and analyzed by AxioVision 4.6 software (Carl Zeiss Microimaging, Thornwood, NY).

### 2.5. Statistical Analysis

All the experiments were performed, at least, three times. Quantitation of data was performed using ImageJ software (NIH, MA). Statistical significance of the data was calculated by QuickCals t-test calculator (GraphPad Software, Inc., CA). Statistical significance of control and treated groups is shown as ^*∗*^p<0.05 (significant), ^*∗∗*^p<0.01 (very significant), and ^*∗∗∗*^p<.001 (highly significant). Statistical significance between control versus TXC, OAG/UV/H_2_O_2_ is shown by ^&^ and ^$^ symbols, respectively, and that of OAG versus OAG+extracts are shown by ^**#**^ and *∗* symbols.

## 3. Results and Discussion

### 3.1. Effect of OAG and TXC on Human Melanoma and Primary Melanocytes

**Figure 1:**
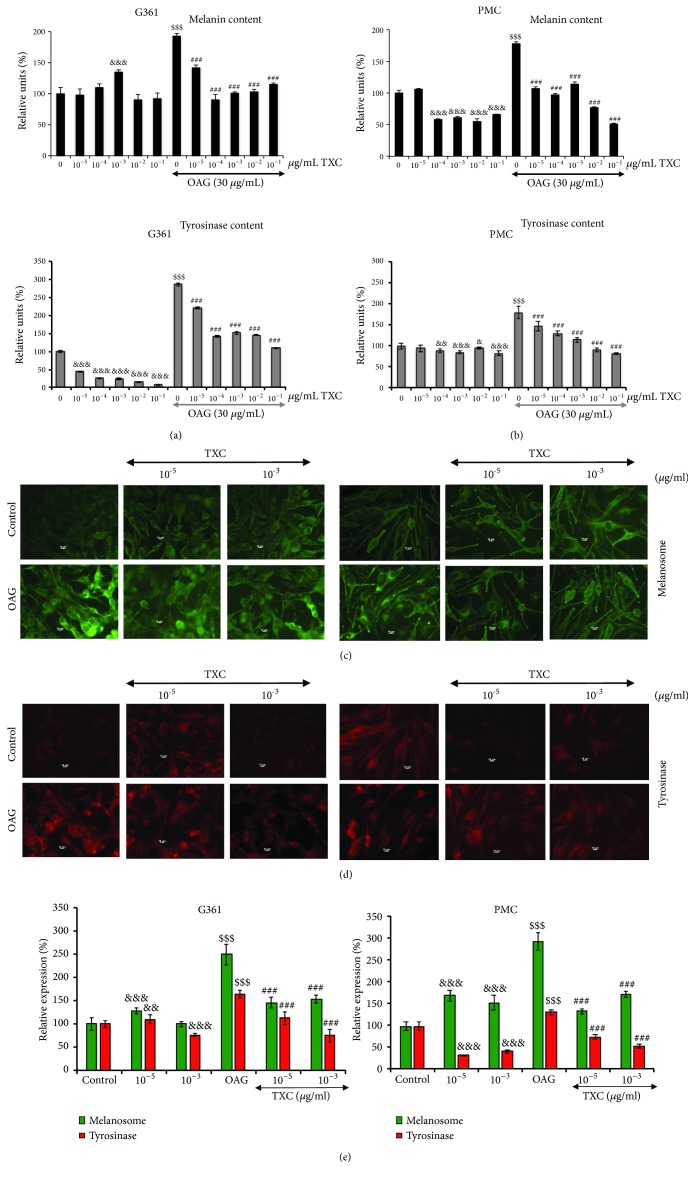
Effect of OAG and TXC on melanin and tyrosinase contents in human melanoma (G361) (a) and primary melanocytes from Caucasian skin (PMC) (b). Cells were treated with OAG for 24 h followed by recovery either in control (OAG) or TXC-supplemented medium as indicated. Melanosome (c) and tyrosinase (d) immunofluorescence signals in control and OAG-treated cells, recovered in either control, or TXC-supplemented medium are shown. (e) Quantitation of the immunofluorescence for melanosome and tyrosinase are shown.

*UV and *OAG have been established as inducer of melanogenesis. We first examined the comparative and dose-dependent response of human melanoma cells (G361) to UV and OAG. As shown in Supplementary Figure 1, both UV and OAG-treated G361 cells showed dose-dependent increase in melanosomes and melanin content. Of note, whereas UV-treated cells showed some toxicity and mild increase in melanogenesis, OAG-treated cells did not show toxicity, rather there was a stronger increase in melanin content, reproducible in several experiments. Based on these data, we adopted OAG for the present study. Cytotoxicity of OAG to human melanoma (G361) and primary melanocytes from Caucasian skin (PMC) was next determined extensively by cell viability assays (data not shown) and nontoxic doses (15-60 *μ*g/ml) were used. The effect of TXC on cultured cells has not been reported so far. We first determined its effect on G361 and PMC. We found that G361 cells well tolerated the treatment at doses from 10^−5^ to 10^-1 ^*μ*g/ml. PMC cells showed some cytotoxicity at doses more than 10^-2 ^*μ*g/ml (Supplementary Figure 2). Nontoxic doses of OAG were used in combination with TXC to examine their combined effect on cell viability. We found that OAG-induced stress was not escalated when cells were recovered in the presence of TXC (nontoxic doses) in the medium (Supplementary Figure 2 and data not shown). Next, we investigated the effect of TXC on melanin formation and tyrosinase expression. G361 cells, upon treatment with TXC, did not show any significant difference in melanin content although the tyrosinase expression showed dose-dependent decrease in OAG-untreated cells (Figures 1(a) and 1(b)). OAG treatment caused 2-3-fold increase in melanin content and tyrosinase expression. Of note, such increase was attenuated when cells were recovered in TXC-supplemented medium. PMC showed similar response. OAG-treated PMC showed increase in melanin and tyrosinase expression and, similar to G361, TXC-treated PMC showed reduction in melanin as well as tyrosinase expression (Figures 1(a) and 1(b)). However, in contrast to G361 that did not show any effect on endogenous melanin content, PMC showed about 30% decrease in melanin when treated with TXC at doses more than 10^-5 ^*μ*g/ml. Tyrosinase expression showed decrease in G361, but not in PMC (Figures 1(a) and 1(b)). The results were endorsed by immunofluorescence assays that showed clear intensification of melanosomes and tyrosinase in OAG-treated (both G361 and PMC) cells and their reduction in response to by TXC treatment (Figures 1(c), 1(d), and 1(e)).

### 3.2. OAG and TXC Induced Changes in Melanogenesis Correlated with Expression of Stress Chaperones

We have earlier identified stress response proteins such as HSP60, Bcl2, Bcl-xL, p53, and mortalin to contribute to OAG-induced increase in melanogenesis [14]. Melanin assays in cells compromised for these proteins by specific shRNA caused reduction in OAG-induced increase in melanin. On the other hand, G361 cells with overexpression of these proteins showed increase in melanin content that occurred independent of that of the tyrosinase expression. The data suggested that these genes may regulate melanogenesis by tyrosinase independent pathways that may range from DNA damage, oxidation, or inflammation or mitochondrial stresses as suggested by other studies [21, 22]. Mitochondrial proteins, prohibitin, and mortalin have been linked to melanogenesis [14, 23–27].

**Figure 2:**
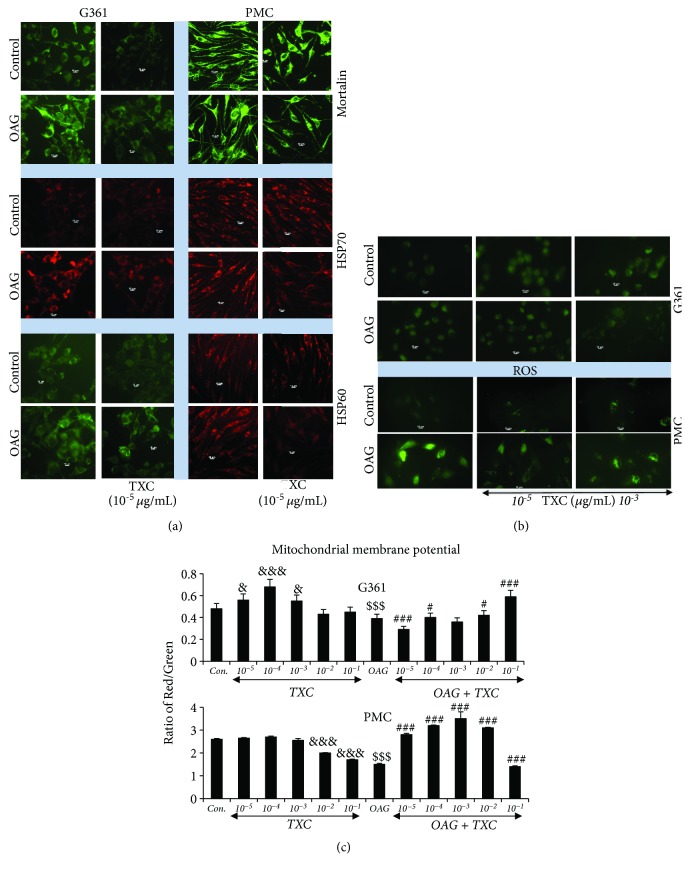
Effect of OAG and TXC on mortalin, HSP70, and HSP60 expressions in human melanoma (G361) and primary melanocytes from Caucasian skin (PMC) as determined by immunocytochemistry using specific antibodies (a). Cells were treated with OAG for 24 h followed by recovery either in control (OAG) or TXC-supplemented medium as indicated. Effect of OAG and TXC on Reactive Oxygen Species (ROS) (b) and mitochondrial membrane potential (c) in human melanoma (G361) and primary melanocytes from Caucasian skin (PMC) as determined by live cell ROS detection and JCI staining, respectively. Cells were treated with OAG for 24 h followed by recovery either in the control (OAG) or TXC-supplemented medium as indicated.

In light of the above reports, we next investigated the effect of TXC on stress response proteins. OAG-treated both G361 and PMC cells showed increase in mortalin, HSP70, and HSP60 supporting their involvement in the process of melanogenesis (Figures 2(a) and 2(b)). Remarkably, TXC-treated cells (both G361 and PMC) showed decrease in OAG-induced upregulated mortalin, HSP70, and HSP60 (Figure 2(a) and Supplementary Figures 3A and 3B). Consistently, oxidative stress as determined by live imaging of Reactive Oxygen Species (ROS) was higher in OAG-treated cells and exhibited attenuation in response to TXC treatment (Figure 2(b) and Supplementary Figure 3C). Mitochondrial membrane potential, determined by JC1 staining, was found to decrease in OAG-treated cells and was protected by TXC treatment (Figure 2(c)) suggesting tight correlation between the oxidative stress and melanogenesis.

### 3.3. Manipulation of Cell Pigmentation by Herbal Extracts-Involved Changes in Expression of Stress Chaperones

**Figure 3:**
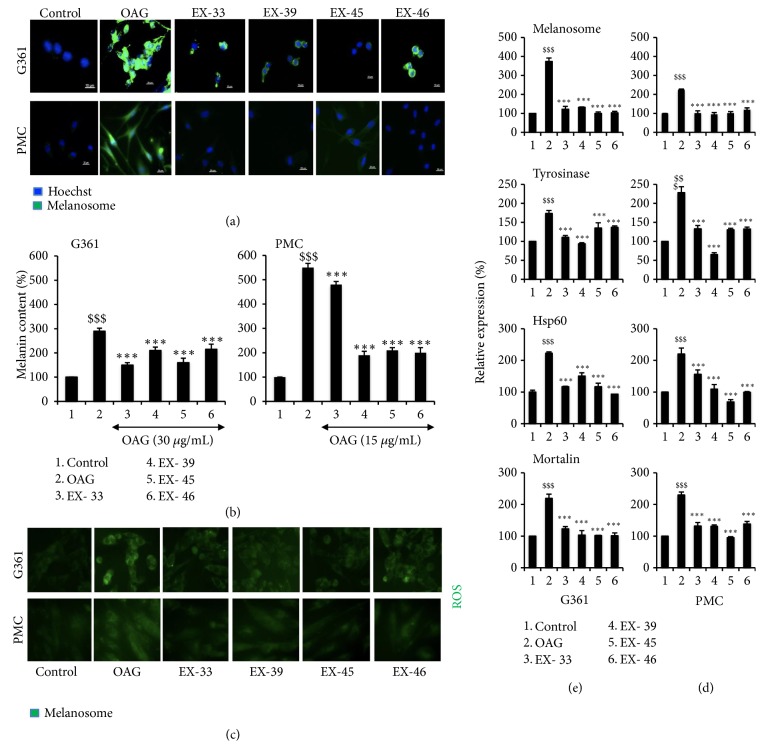
Effect of selected natural extracts on OAG-induced increase in melanosomes (a) and melanin content (b) in human melanoma (G361) and primary melanocytes from Caucasian skin (PMC). Cells were treated with OAG for 24 h followed by recovery either in control (OAG) or extract (EX-33, EX-39, EX-45, and EX-46) supplemented medium as indicated. Increase in OAG-induced ROS and its reversal in the presence of extracts are shown (c). Increase in melanosomes, Tyrosinase, HSP60, and mortalin in OAG-treated human melanoma (G361) (d) and primary melanocytes from Caucasian skin (PMC) (e) and their reversal in response to treatment with extract (EX-33, EX-39, EX-45, and EX-46) are shown. Quantitation of the data obtained from three independent experiments is shown with each panel.

**Figure 4:**
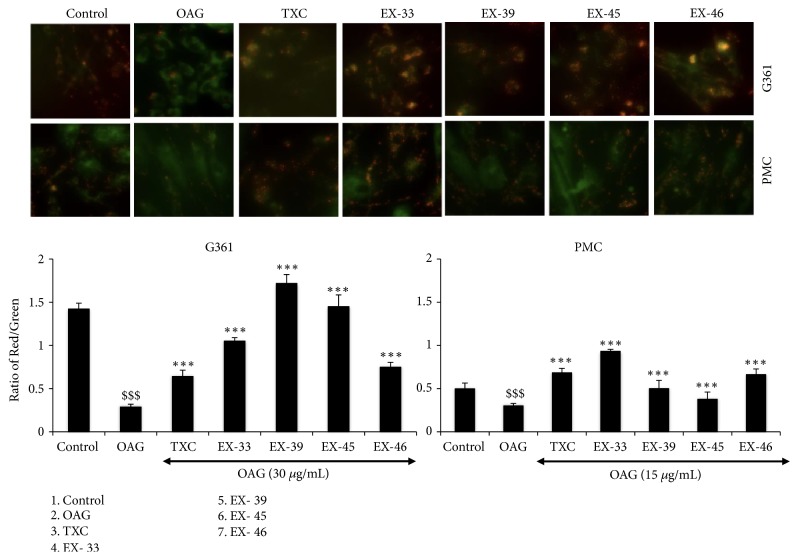
Effect of selected natural extracts on OAG-induced decrease in mitochondrial membrane potential and their reversal in the presence of TXC and extracts in both human melanoma (G361) and primary melanocytes (PMC) is shown.

**Figure 5:**
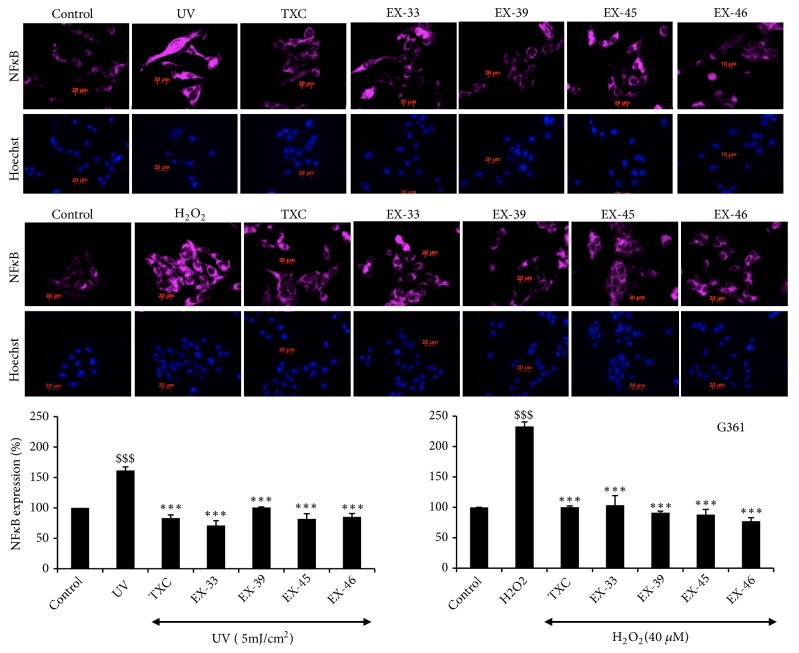
Effect of selected natural extracts on oxidative (UV and H_2_O_2_) induced NFkB expression. Whereas both UV and H_2_O_2_ caused upregulation of NFkB expression, its reversal in cells treated with TXC and extracts (EX-33, EX-39, EX-45, and EX-46) was observed. Quantitation of the data obtained from three independent experiments is shown.

In order to further investigate the relation of melanogenesis with stress signaling proteins, we recruited 40 herbal extracts/purified ingredients (Supplementary Table 1). Effect of these extracts on OAG-induced melanogenesis was determined by melanin assays in G361 cells. The extracts that showed no toxicity and caused reduction in OAG-induced melanin in G361 cells were further analyzed to examine the oxidative and stress signaling. DMSO (solvent used for the extracts) was used as a control and did not affect OAG-induced melanogenesis (Supplementary Figure 3D). Four of the forty extracts, EX-33 (*Glycyrrhiza glabra*; ethanol extract), EX-39 (*Prunus mume*; aqueous extract), EX-45 (*Scutellaria baicalensis *Georgi*; Aqueous extract*), and EX-46 (*Camellia sinensis; Aqueous extract*), were selected for further analyses. Biochemical and immunofluorescence assays revealed that these 4 extracts were capable of inhibiting OAG-induced increase in melanin, in both G361 and PMC cells (Figures 3(a) and 3(b) and Supplementary Figure 3E). Molecular analyses of ROS in control, OAG, and extract-treated cells showed increase that was consistent with the increase in melanin. Remarkably, the extract-treated cells showed decrease in the level of in ROS in both G361 and PMC (Figure 3(c) and Supplementary Figure 3F). We further extended the analyses on stress proteins. As shown in Figures 3(d) and 3(e) and Supplementary Figures 4 and 5, respectively, OAG-induced increase in melanosomes, tyrosinase was accompanied by increase in stress protein mortalin in both G361 and PMC endorsing the relation of melanogenesis with mortalin-signaling. We next examined the status of mortalin expression when melanin is decreased in response to extract treatment. Interestingly, the level of mortalin also attenuated suggesting their tight relation. Coincidently, similar changes were observed in HSP60, another mitochondrial stress chaperone, suggesting that the two proteins may link melanogenesis and stress response. Consistent with these changes, analysis of mitochondrial membrane potential (Ω) in control and extract-treated cells showed its attenuation in OAG-treated cells and recovery by extracts (Figure 4). OAG-treated cells showed remarkable decease in Ω. TXC and extract-treated cells showed recovery in Ω suggesting that they possess anti-OAG stress properties. In light of the information that TXC possesses anti-inflammatory properties, we next compared the selected compounds for anti-inflammation effect. The cells were subjected to UV and H_2_O_2_, known inducers of DNA damage, inflammation, and pigmentation [28, 29]. As expected, UV- and H_2_O_2_-exposed cells showed induction of NF*κ*B expression (Figure 5). Of note, TXC and the extracts (EX-33, EX-39, EX-45, or EX-46) treated cells showed ~50% decrease in NF*κ*B. In these premises, further chemical characterizations of extracts are warranted for their potential use in preventive and therapeutics in disease and cosmetics treatments.

Melanogenesis is a stress response. It has been well-established that exposure to UV, oxidative stresses, and others cause induction of melanogenic proteins and darkening of skin [28, 29]. Level of constitutive HSP70 has been shown to inversely relate to melanin production and hence was used to identify the hypopigmenting reagents [30–32]. However, induction of melanogenesis by IBMX as determined by expression of MITF and tyrosinase activity was not suppressed by HSP70 overexpression. These findings have suggested the involvement of other proteins/factors [33]. By loss-of-function screening, we earlier identified mtHSP70/mortalin to be involved in the process of oxidative stress-induced melanogenesis [14]. Upregulation of mortalin was associated with increase in melanin. On the other hand, whereas mortalin-overexpressing cells showed increase in melanogenesis, mortalin-compromised cells showed decrease. Consistent to these findings, hyperpigmented skin showed high level of mortalin expression, its knockdown caused depigmentation [34]. Other mitochondrial proteins shown to regulate melanogenesis include prohibitin [25], phosphorylated CREB, ATP5B, and mitochondrial F1 complex [26]. Induction of melanogenesis has also been correlated with increase in intracellular ATP levels suggesting the involvement of mitochondria [26]. It was shown that mitochondria physically contact melanosomes during the process of melanogenesis. It is achieved through fibrillar bridges involving mitofusin 2 that also connects mitochondria to ER [27]. Knockdown of mitofusin 2 was shown to cause reduction in mitochondria-melanosome contacts leading to atypical melanogenesis. The mitochondrial biogenesis is determined by import of several precursor proteins synthesized in cytoplasm and translocated across the mitochondrial inner membrane by the action of an essential mitochondrial inner membrane translocase motor protein complex, Tim44-complex. The complex has been shown to interact with mitochondrial chaperones, mtHSP70/mortalin, and HSP60 that interact with each other and perform several house-keeping functions [35]. Abnormalities in the functions of these chaperones have been associated with mitochondrial fragmentation [36–38] and anomalous melanogenesis [14, 27, 34]. Pan et al. [39] showed that the UV-induced melanin synthesis in dopaminergic neurons was associated with occurrence of Parkinson's disease (PD) and melanoma. Alpha-synuclein is expressed in brain and skin and shown to interact with tyrosinase that regulates biosynthesis of melanin and dopamine. Interestingly, mortalin has been shown to interact with alpha-synuclein and contributes to PD pathogenesis [40–45]. In light of this information, it was deliberated that natural and synthetic agents that modify stress-induced melanogenesis may also be useful for prevention or treatment of not only the pigmentation-related disorders but also other diseases including Parkinson's and melanoma. Of note, we found that TXC and five natural extracts have potential to modulate stress, melanogenesis, and inflammation signaling. Since stress chaperone mortalin has been functionally linked to several pathologies including cancer, hyperpigmentation, mitochondrial function, and Parkinson's disease, the selected extracts that affect mortalin warrant further studies for their use in cosmetics and disease therapeutics.

## Data Availability

The data used to support the findings of this study are available from the corresponding author upon request.

## Supplementary Materials

Supplementary Materials Supplementary Figure 1: effect of UV and OAG on melanin content as determined by melanosome staining in human melanoma (G361) cells. Cells were treated with either UV or OAG for 24 h followed by recovery for 24-48 h. Quantitation of melanin content showed dose-dependent response and strong effect of OAG. Supplementary Figure 2: effect of OAG and TXC on viability of human melanoma (G361) (A) and primary melanocytes from Caucasian skin (PMC) (B). Cells were treated with OAG for 24 h followed by recovery for 24-48 h in either control or TXC (as indicated) supplemented media. Cell morphology was recorded under the microscope. Supplementary Figure 3: quantitation of the effect of OAG and TXC on expression of stress proteins, mortalin, HSP70, and HSP60, in human melanoma (G361) (A) and primary melanocytes from Caucasian skin (PMC) (B) as determined by immunocytochemistry using specific antibodies. Quantitation of the effect of OAG and TXC on Reactive Oxygen Species (ROS) in G361 and PMC cells (C). Effect of DMSO, TXC, and extracts on OAG-induced increase in melanin, whereas DMSO was neutral; TXC and the extracts caused decrease in OAG-induced increase in melanin (D). Quantitation of the effect of OAG and extracts on melanin content of G361 and PMC shown in Figure 3A (E). Quantitation of the effect of OAG and extracts on level of ROS in G361 and PMC shown in Figure 3C (F). Supplementary Figure 4: immunofluorescence images of G361 cells treated with OAG followed by recovery in extract supplemented medium. Quantitation of the data and statistical significance are shown in Figure 3D. Supplementary Figure 5: immunofluorescence images of PMC cells treated with OAG followed by recovery in extract supplemented medium. Quantitation of the data and statistical significance is shown in Figure 3E. Supplementary Table 1: list of extracts and ingredients tested for their effect on OAG-induced melanogenesis.
