# Supplementary material for: Modulation of Diacylglycerol-Induced Melanogenesis in Human Melanoma and Primary Melanocytes: Role of Stress Chaperone Mortalin
Source: Evid Based Complement Alternat Med. 2019 Apr 14;2019:9848969. doi: 10.1155/2019/9848969 (PMC6487102; doi:10.1155/2019/9848969)
Supplement: Supplementary Materials — Supplementary Figure 1: effect of UV and OAG on melanin content as determined by melanosome staining in human melanoma (G361) cells. Cells were treated with either UV or OAG for 24 h followed by recovery for 24-48 h. Quantitation of melanin content showed dose-dependent response and strong effect of OAG. Supplementary Figure 2: effect of OAG and TXC on viability of human melanoma (G361) (A) and primary melanocytes from Caucasian skin (PMC) (B). Cells were treated with OAG for 24 h followed by recovery for 24-48 h in either control or TXC (as indicated) supplemented media. Cell morphology was recorded under the microscope. Supplementary Figure 3: quantitation of the effect of OAG and TXC on expression of stress proteins, mortalin, HSP70, and HSP60, in human melanoma (G361) (A) and primary melanocytes from Caucasian skin (PMC) (B) as determined by immunocytochemistry using specific antibodies. Quantitation of the effect of OAG and TXC on Reactive Oxygen Species (ROS) in G361 and PMC cells (C). Effect of DMSO, TXC, and extracts on OAG-induced increase in melanin, whereas DMSO was neutral; TXC and the extracts caused decrease in OAG-induced increase in melanin (D). Quantitation of the effect of OAG and extracts on melanin content of G361 and PMC shown in Figure 3A (E). Quantitation of the effect of OAG and extracts on level of ROS in G361 and PMC shown in Figure 3C (F). Supplementary Figure 4: immunofluorescence images of G361 cells treated with OAG followed by recovery in extract supplemented medium. Quantitation of the data and statistical significance are shown in Figure 3D. Supplementary Figure 5: immunofluorescence images of PMC cells treated with OAG followed by recovery in extract supplemented medium. Quantitation of the data and statistical significance is shown in Figure 3E. Supplementary Table 1: list of extracts and ingredients tested for their effect on OAG-induced melanogenesis. [file 9848969.f1.pdf]

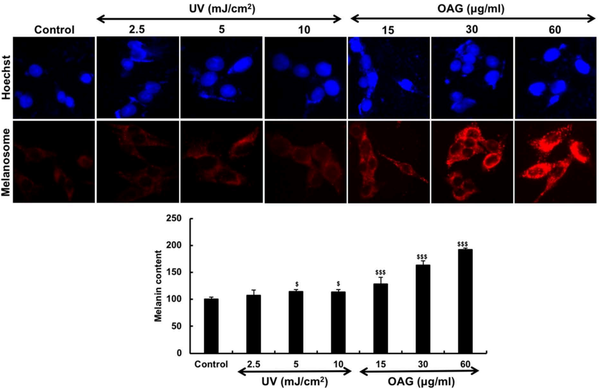

Supplementary Fig. 1

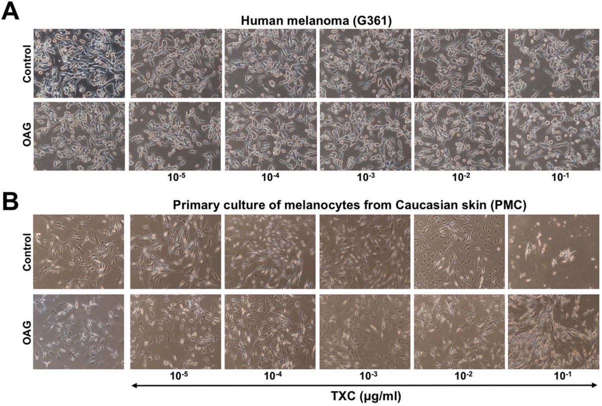

Supplementary Fig. 2

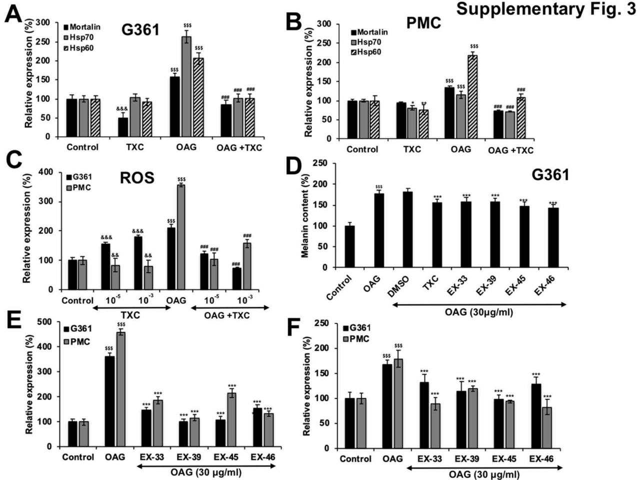

Supplementary Fig. 3
A
G361
Mortalin
Hsp70
Hsp60
Relative expression (%)
Control
TXC
OAG
OAG +TXC
B
PMC
Mortalin
Hsp70
Hsp60
Relative expression (%)
Control
TXC
OAG
OAG +TXC
C
ROS
G361
PMC
Relative expression (%)
Control
10-5
10-3
OAG
10-5
10-3
TXC
OAG +TXC
D
G361
Melanin content (%)
Control
OAG
DMSO
TXC
EX-33
EX-39
EX-45
EX-46
OAG (30µg/ml)
E
G361
PMC
Relative expression (%)
Control
OAG
EX-33
EX-39
EX-45
EX-46
OAG (30 µg/ml)
F
G361
PMC
Relative expression (%)
Control
OAG
EX-33
EX-39
EX-45
EX-46
OAG (30 µg/ml)

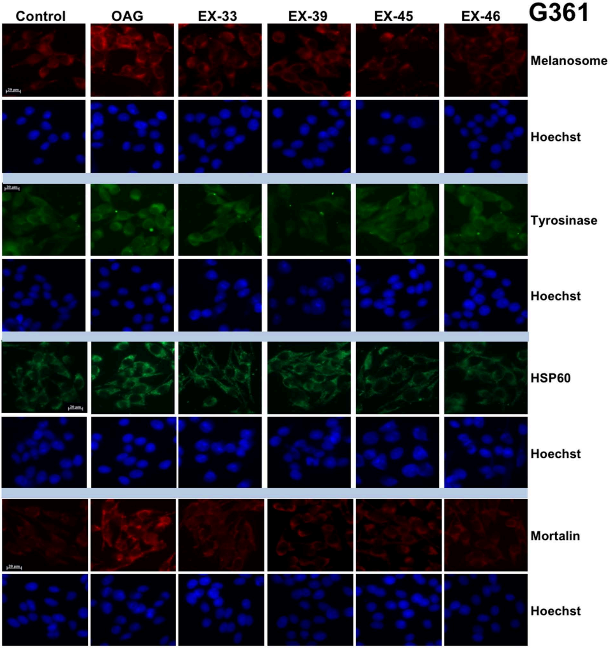

Supplementary Fig. 4

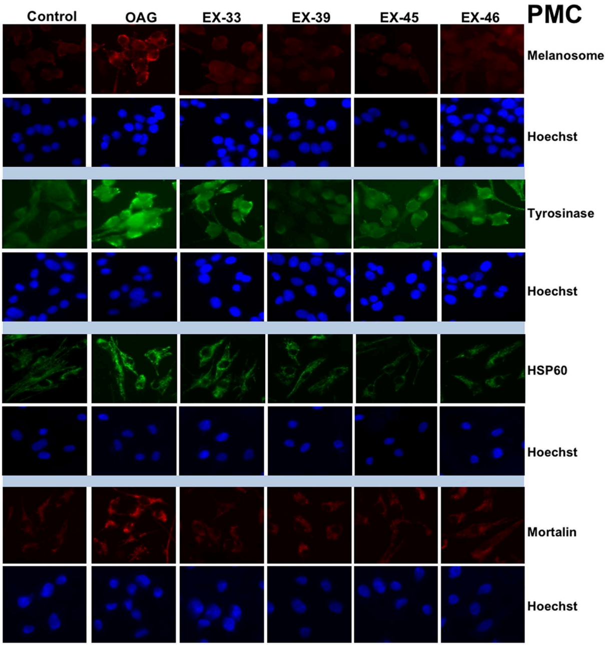

Supplementary Fig. 5

**Supplementary Table 1. Extracts and Ingredients Tested for Their Effect on OAG-induced Melanogenesis**					
Sr. Number	Reagent ID	Name	Sr. Number	Reagent ID	Name
1	R-01	Hyperoside	21	R-21	Embelin
2	R-02	Quercertin 4'-glucoside	22	R-22	Trans-4-(Aminomethyl) cyclohexanecarboxylic acid hexadecyl ester hydrochloride (TXC)
3	R-03	Licochalcone A	23	R-23	Tetraethylene glycol (TTEG)
4	R-04	Xanthohumol	24	R-24	Pentaethylene glycol (PEG)
5	R-05	Luteolin	25	R-25	Triethylene glycol dimethyl ether (TEGdmE)
6	R-06	Tectorigenin	26	R-26	Triethylene glycol monobutyl ether
7	R-07	5,7-Dimethoxyflavone	27	R-27	Creatine
8	R-08	Tectorigenin 7-o-xylosylglucoside	28	R-28	Epolactaene
9	R-09	Daidzein	29	R-29	Trifluoromethoxy carbonylcyanide phenylhydrazone
10	R-10	Soyasaponin I	30	R-30	Tetraethylene glycol dimethyl ether
11	R-11	Soyasapogenol A	31	R-31	Triethylene glycol dimethacrylate
12	R-12	Ginkgolide A	32	R-32	Tetraethylene glycol dimethacrylate
13	R-13	Glycyrrhizinic acid	33	EX-33	*Glycyrrhiza glabra* extract
14	R-14	Deacylgymnemic acid	34	R-34	Tranexamic acid (TXA)
15	R-15	Ganoderic acid A	35	EX-35	Tea flower extract
16	R-16	Corosolic acid	36	EX-36	Mushroom extract
17	R-17	Bilobalide	37	R-37	Rucinol
18	R-18	Verbascosid	38	EX-39	*Prunus mume* extract
19	R-19	Geniposide	39	EX-45	*Scutellaria baicalensis georgi* extract
20	R-20	6-Gingerol	40	EX-46	*Camellia sinensis* extract
